# Supplementary material for: Kit ligand has a critical role in mouse yolk sac and aorta–gonad–mesonephros hematopoiesis
Source: EMBO Rep. 2018 Aug 30;19(10):e45477. doi: 10.15252/embr.201745477 (PMC6172468; doi:10.15252/embr.201745477)
Supplement: Supplementary file 1 — Appendix [file EMBR-19-e45477-s001.pdf]

APPENDIX

TABLE OF CONTENTS

		Page no.
**Appendix Figure S1**	Flow cytometry and imaging analysis of tissue macrophages	1
**Appendix Figure S2**	Engraftment analysis of wild type/heterozygous and *Sl/Sl* AGM HSCs	2
**Appendix Figure S3**	Multiplex qRT-PCR of wild type and *Sl/Sl* embryonic hematopoietic progenitors	3
**Appendix Figure Legends**		4
**Appendix Table S1**	Genotyping primers	7
**Appendix Table S2**	Antibodies used for immunofluorescence staining	8
**Appendix Table S3**	Antibodies and viability stains used for flow cytometric analysis and cell sorting	9
**Appendix Table S4**	Taqman probes used for classic and multiplex qRT-PCR analysis.	12
**Appendix Table S5**	List of genes used in multiplex qRT-PCR analysis and references.	13
**Supplemental references**		14

Appendix Figure S1

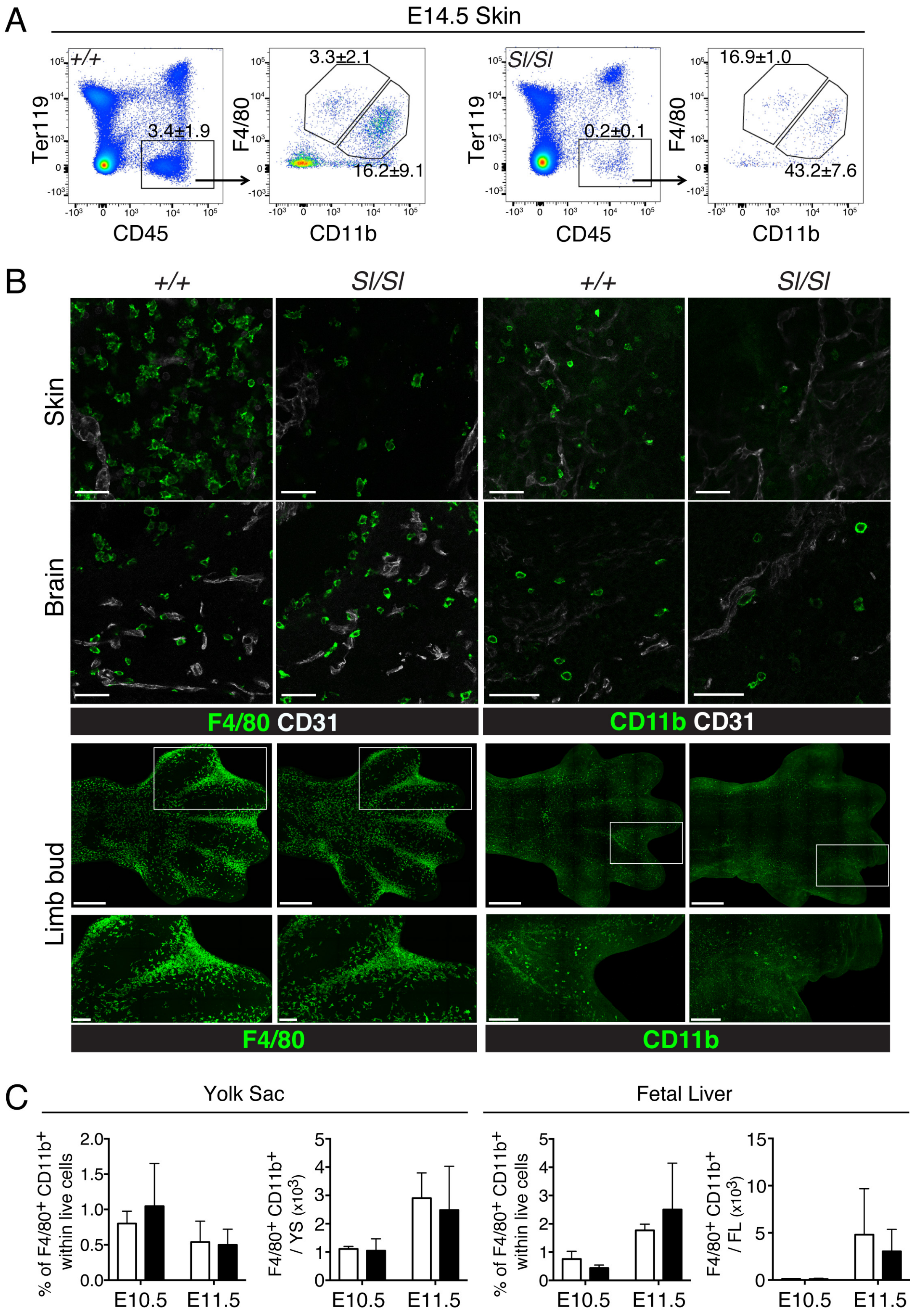

Appendix Figure S2

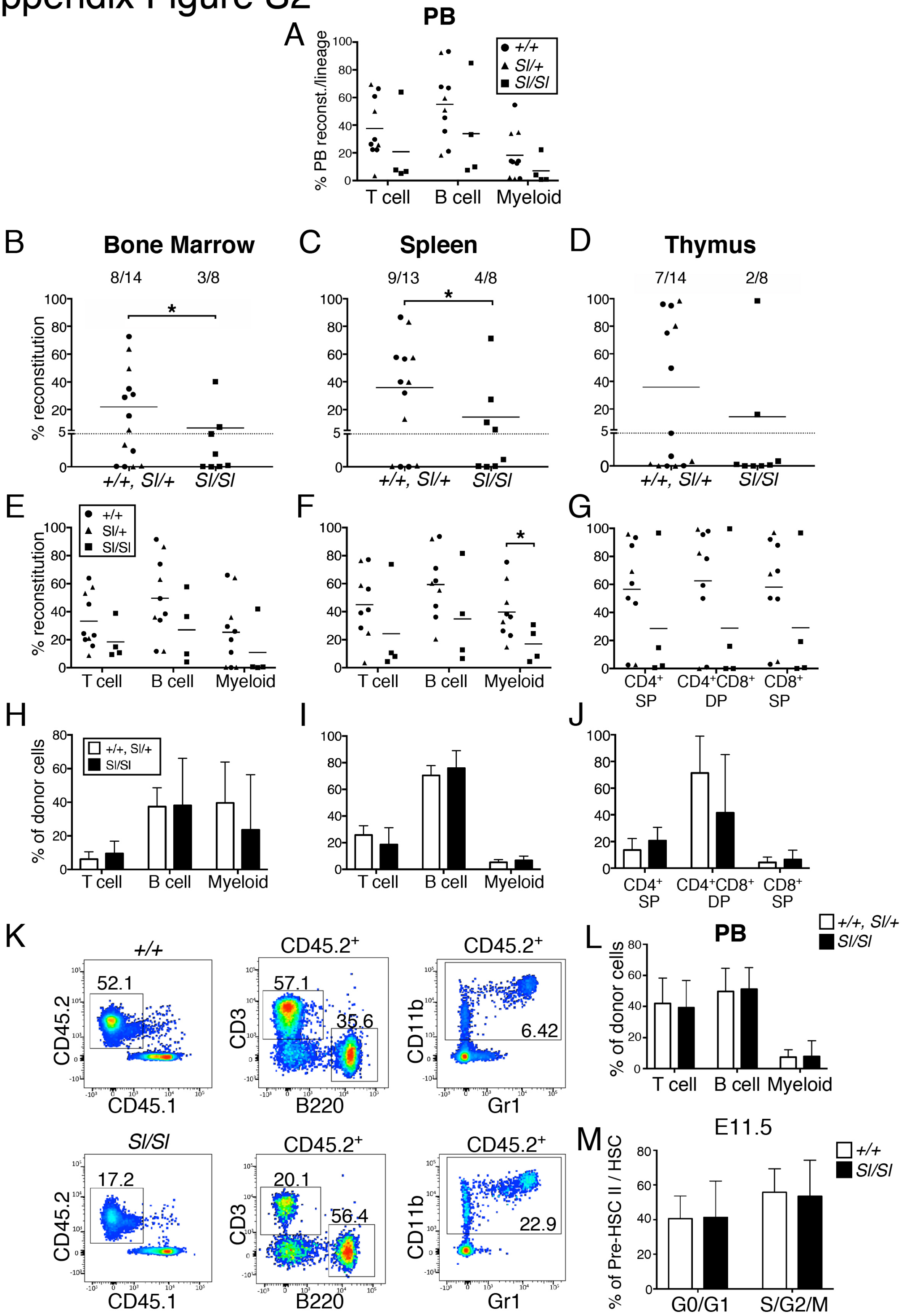

Appendix Figure S3

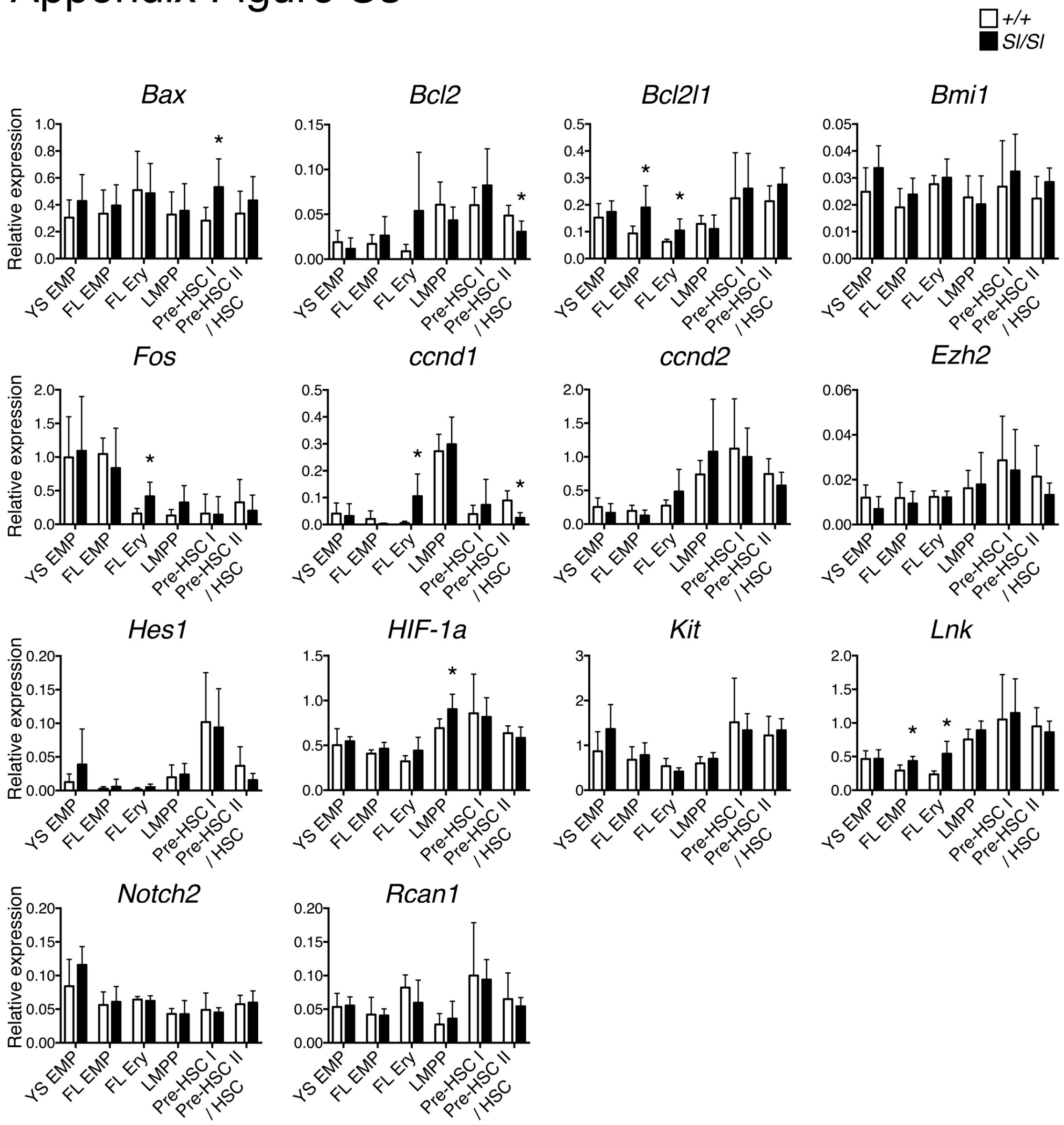

APPENDIX FIGURE LEGENDS

Appendix Figure S1. Flow cytometry and imaging analysis of tissue macrophages

A. Representative flow cytometry analysis of macrophages in wild type and *Sl/Sl* E14.5 skin dissected from the back. Cells were gated as shown, with percentages in individual gates as indicated. Data are the mean (±SD) of 4 biological replicates for either genotype, with each replicate consisting of individual or pooled samples (from up to 3 embryos). A total of 8 wild type and 5 *Sl/Sl* embryos were analyzed over 2 independent experiments.

B. Confocal whole-mount immunofluorescence analysis of macrophages in back skin, brain and forelimb buds of wild type and *Sl/Sl* E14.5 embryos. For skin and brain, representative images of single 2.5μm-thick optical slices are shown. Limb bud images are maximum intensity 3D projections from 300μm-thick Z stacks. Boxed area is magnified. Scale bars: 50μm (skin, brain), 300μm (limb buds), 100μm (magnified area). A total of 2 wild type and 2 *Sl/Sl* embryos were analyzed.

C. Flow cytometry analysis of macrophages in wild type and *Sl/Sl* E10.5-E11.5 yolk sac and fetal liver. Data are the mean (±SD) of 4 wild type (32-35 sp) or 3 *Sl/Sl* (34-35 sp) replicates, analyzed over 2 independent experiments. E11.5 data are the mean (±SD) of 4 wild type (10-13 ts) or 3 *Sl/Sl* (10-13 ts) replicates, analyzed over 3 independent experiments. Embryos were analyzed individually.

Appendix Figure S2. Engraftment analysis of wild type/heterozygous and *Sl/Sl* AGM HSCs

A. Lineage reconstitution in peripheral blood (PB) of individual mice transplanted with wild type or *Steel* mutant AGM cells. Percentages of donor-derived reconstitution in the T ($CD3^+$), B ($B220^+$) and myeloid ($CD11b^+$) lineages are shown, 16 weeks post-transplant. p=0.239 (T cells), p=0.187 (B cells), p=0.176 (Myeloid), two-tailed Mann-Whitney's U test.

B. Repopulation analysis of irradiated CD45.1 syngeneic mice transplanted with 1e.e. of wild type (+/+), Sl/+ or *Sl/Sl* E11.5 $CD45.2^+$ AGM+VU cells. Graph shows the percentage of chimerism in bone marrow represented as the percentage of donor $CD45.2^+$ cells on total $CD45^+$ cells, 16 weeks after transplant. N=14 (+/+ or *Sl*/+), N=8 (*Sl/Sl*) recipients analyzed; 4 independent experiments. Tail somite range: 9-17 (+/+, *Sl*/+); 10-17 (*Sl/Sl*). p=0.036, two-tailed Mann-Whitney's U test.

C. Repopulation analysis as in (B) showing the percentage of chimerism in spleen. p=0.03, one-tailed Mann-Whitney's U test; p=0.059, two-tailed Mann-Whitney's U test.

D. Repopulation analysis as in (B) showing the percentage of chimerism in thymus. p=0.928, two-tailed Mann-Whitney's U test.

E. Results from flow cytometric analysis of bone marrow of repopulated mice 16 weeks after transplant, showing the percentage of donor-derived T cells ($CD3^+$), B cells ($B220^+$) and myeloid cells ($CD11b^+$). p=0.141 (T cells), p=0.187 (B cells), p=0.453 (Myeloid), two-tailed Mann-Whitney's U test.

F. Results from flow cytometric analysis of spleen of repopulated mice 16 weeks after transplant, showing the percentage of donor-derived T cells ($CD3^+$), B cells ($B220^+$) and myeloid cells ($CD11b^+$). p=0.26 (T cells), p=0.198 (B cells), p=0.049 (Myeloid), two-tailed Mann-Whitney's U test.

G. Results from flow cytometric analysis of thymus of repopulated mice 16 weeks after transplant, showing the percentage of donor-derived $CD4^+$ single positive (SP), $CD4^+$ $CD8^+$ double positive (DP) and $CD8^+$ SP. p=0.33 ($CD4^+$ SP), p=0.414 ($CD4^+$ $CD8^+$ DP), p=0.26 ($CD8^+$ SP), two-tailed Mann-Whitney's U test.

H. Lineage analysis of repopulated mice 16 weeks after transplant, showing the percentage of T cells ($CD3^+$), B cells ($B220^+$) or myeloid cells ($CD11b^+$) within donor-derived $CD45.2^+$ cells in bone marrow. Data are mean (±SD).

I. Lineage analysis of repopulated mice 16 weeks after transplant, showing the percentage of T cells ($CD3^+$), B cells ($B220^+$) or myeloid cells ($CD11b^+$) within donor-derived $CD45.2^+$ cells in spleen. Data are mean (±SD).

J. Lineage analysis of repopulated mice 16 weeks after transplant, showing the percentages of $CD4^+$ single positive (SP), $CD4^+$ $CD8^+$ double positive (DP) and $CD8^+$ SP within donor-derived $CD45.2^+$ in thymus. Data are mean (±SD).

K. Flow cytometry analysis of peripheral blood at 16 weeks post transplant showing representative examples of recipients reconstituted with wild type (+/+) or *Sl/Sl* E11.5 $CD45.2^+$ AGM+VU cells.

L. Percentage of T cells ($CD3^+$), B cells ($B220^+$) or myeloid cells ($CD11b^+$) among donor-derived PB cells of mice transplanted with wildtype or *Sl/+*, or with *Sl/Sl*, AGM+VU, 16 weeks post-transplant. A total of 14 recipients were transplanted with wild type or *Sl/+* cells and 8 with *Sl/Sl* cells, over 4 independent experiments. Data are mean (±SD).

M. Cell cycle status of pre-HSC type II/HSCs ($Ter119^-$ $VE\text{-}Cadherin^+$ Kit^+ $CD41^+$ $CD45^+$) in E11.5 wild type and *Sl/Sl* AGM+VU. Percentages of cells in G0/G1 and S/G2/M were analyzed by flow cytometry on the basis of BrdU and 7-AAD incorporation. Data are the mean (±SD) of 3 independent experiments. A total of 6 wild type and 5 *Sl/Sl* embryos (12-17 tail sp) were analyzed individually. No difference was observed in cell cycle status between wild type and mutant pre-HSC type II/HSCs.

Appendix Figure S3. Multiplex qRT-PCR of wild type and *Sl/Sl* embryonic hematopoietic progenitors

Multiplex Fluidigm qRT-PCR analysis on pre-HSC type I (Ter119^{-} VE-Cadherin^{+} Kit^{+} CD41^{+} CD45^{-}), pre-HSC type II/HSC (Ter119^{-} VE-Cadherin^{+} Kit^{+} CD41^{+} CD45^{+}), YS and FL EMP (Ter119^{-} Kit^{+} CD41^{+} CD16/32^{+}), FL Early E (Ter119^{-} Kit^{+} CD41^{-}) and peripheral blood LMPP (Lin^{-} CD19^{-} B220^{-} CD45^{+} Kit^{+} Flt3^{+} IL7Rα^{+}), from E11.5 wild type (n=4 biological replicates; total number of embryos: 9; 11-17ts) and *Sl/Sl* (n=5 biological replicates; total number of embryos: 9; 11-17ts). Genes are shown in alphabetical order. See Appendix Table S4 for full gene names and the list of Taqman assays used here. See also Appendix Table S5 for literature references for these genes. Note: *Bax* and *Bcl2* expression in (pre-)HSCs is also included in Figure 5.

Appendix Table S1. Genotyping primers

Kitl-tdTomato [1]	TomatoF: CACCTCCCACAACGAGGACTACACC<br>KitlR: TCTCACACCACGCCTGTCTCTCC<br>KitL 5' UTR F: GGGCTTCATTTGCTGTCTGTCACC
Steel [2,3]	SL-1: CGGGGTTTATGAGGGTAGGA<br>SL-2: TTGGGCCTGTGTGACAAACT<br>SL-3: GGGGCACTTGGGTACATTC
23GFP	GFP1: GACGTGAACGGCCACAAGTTCA<br>GFP2: GTGGCGGATCTTGAAGTTCACC
Tie2-Cre [4]	Cre-Fw: CGTTTTCTGAGCATACCTGGA<br>Cre-Rv: ATTCTCCCACCGTCAGTACG
$Kitl^{flox}$ [5]	Kitl_flox_Fw: CGAGGTAGGGGAAAAGAACC<br>Kitl_flox_Rv: GGATCTTCCCAGAGGTTGGA

Appendix Table S2. Antibodies used for immunofluorescence staining

Antigen	Clone	Host	Species reactivity	Supplier
Primary Antibodies				
α-SMA (Cy3 conjugate)	1A4	Mouse	Mouse, Rat, Human	Sigma-Aldrich
c-Kit	2B8	Rat	Mouse, Pig	eBioscience
cCasp9	5A1E	Rabbit	Mouse, Rat, Human	Cell Signaling
CD11b	M1/70	Rat	Mouse, Human	BD Pharmingen
CD31	-	Goat	Mouse	R&D Systems
CD31	MEC13.3	Rat	Mouse	BD Pharmingen
Dlk1	24-11	Rat	Mouse	MBL
F4/80	CI:A3-1	Rat	Mouse	Bio-Rad
GFP	-	Rabbit	Tag	ThermoFisher
PDGFR-β	APB5	Rat	Mouse	eBioscience
pHH3	-	Rabbit	Mouse, Rat, Human	Abcam
Phospho-p44/42 MAPK (Erk1/2) (Thr202/Tyr204)	D13.14.4E	Rabbit	Mouse, Rat, Human, others	Cell Signaling
Phospho-Akt (Ser473)	D9E	Rabbit	Mouse, Rat, Human, others	Cell Signaling
RFP/tdTomato	-	Rabbit	Tag	Rockland
Runx1	EPR3099	Rabbit	Mouse, Rat, Human	Abcam
VE-Cadherin	BV13	Rat	Mouse	eBioscience
Secondary Antibodies				
IgG (H+L) Alexa Fluor 488	-	Donkey	Rabbit	ThermoFisher
IgG (H+L) Alexa Fluor 488	-	Donkey	Goat	ThermoFisher
IgG (H+L) Alexa Fluor 488	-	Donkey	Rat	ThermoFisher
IgG (H+L) Alexa Fluor 555	-	Donkey	Goat	ThermoFisher
IgG (H+L) Alexa Fluor 555	-	Donkey	Rabbit	ThermoFisher
IgG (H+L) Alexa Fluor 555	-	Goat	Rat	ThermoFisher
IgG (H+L) Alexa Fluor 647	-	Donkey	Goat	ThermoFisher
IgG (H+L) Alexa Fluor 647	-	Chicken	Rat	ThermoFisher

Appendix Table S3. Antibodies and viability stains used for flow cytometric analysis and cell sorting

Antibody/Dye	Clone	Supplier	Application(s)
7-aminoactinomycin D (7-AAD)	-	Sigma	Viability dye
Annexin V FITC	-	BD	E11.5 Annexin V staining
B220 Biotin	RA3-6B2	BD	E12.5-E14.5 FL Erythroid staining lineage cocktail
B220 PE-Cy5	RA3-6B2	BD	Repopulation analysis, Lineage cocktail for E12.5-E14.5 FL LSK staining, Lineage cocktail for Fluidigm sorting (LMPP staining)
B220 PE-Cy7	RA3-6B2	BD	Repopulation analysis, E11.5 LMPP staining
BrdU FITC	-	BD	E11.5 BrdU staining
CD11b APC	M1/70	BioLegend	E14.5 macrophage staining
CD11b Biotin	M1/70	BD	E12.5-E14.5 FL Erythroid staining lineage cocktail
CD11b BV421	M1/70	BD	Repopulation analysis
CD11b PE-Cy5	M1/70	BioLegend	Lineage cocktail for BM LSK reconstitution analysis
CD11b PE-Cy7	M1/70	BD	E11.5 macrophage staining
CD150 PE-Cy7	mShad150	eBioscience	BM LSK reconstitution analysis, E12.5-E14.5 FL LSK staining
CD16/32 APC	93	eBioscience	E11.5 BrdU, E11.5 Annexin V stainings, E9.5 EMP analysis, E11.5 YS-FL EMP analysis, E10-E11.5 EMP Blood analysis, E11.5 Fluidigm sorting
CD19 PE-Cy5	eBio1D3	eBioscience	Lineage cocktail for BM LSK reconstitution analysis, Lineage cocktail for E12.5-E14.5 FL LSK staining, Lineage cocktail for E12.5 FL analysis, Lineage cocktail for Fluidigm sorting (LMPP staining)
CD19 PE-Cy7	1D3	BD	E11.5 LMPP staining
CD3 Biotin	145-2C11	BD	E12.5-E14.5 FL Erythroid staining lineage cocktail
CD3e PE	145-2C11	BD	Repopulation analysis
CD3e APC	145-2C11	BD	E11.5 LMPP staining
CD3e PE-Cy5	145-2C11	BioLegend	Lineage cocktail for BM LSK reconstitution analysis, Lineage cocktail for E12.5-E14.5 FL LSK staining, Lineage cocktail for E12.5 FL analysis, Lineage cocktail for Fluidigm sorting (LMPP staining)
CD4 PE	RM4-5	BD	Repopulation analysis
CD4 PE-Cy5	RM4-5	BD	Lineage cocktail for BM LSK reconstitution analysis, Lineage cocktail for E12.5 FL analysis
CD41 ef450	MWReg30	eBioscience	E11.5 BrdU, E11.5 Annexin V stainings, E11.5 FL analysis, E10-E11.5 EMP Blood analysis, E11.5 Fluidigm sorting
CD41 PE	MWReg30	BD	E10.5-E11.5 AGM Pre-HSC staining, E11.5 RNA-Seq and Fluidigm sorting, E9.5 EMP analysis, E11.5 YS-FL EMP analysis
CD41 PE-Cy7	MWReg30	eBioscience	E12.5-E14.5 FL Erythroid staining lineage cocktail, E10.5 FL analysis
CD44 APC-ef780	IM7	eBioscience	E11.5 FL analysis, E10-E11.5 EMP Blood analysis

CD45 AF700	30-F11	BioLegend	E11.5 LMPP staining
CD45 APC-ef780	30-F11	BD	E10.5-E11.5 AGM Pre-HSC staining, E11.5 RNA-Seq and Fluidigm sorting, E14.5 macrophage staining
CD45 ef450	30-F11	eBioscience	E11.5 macrophage staining
CD45 PE	30-F11	BD	E11.5 BrdU, E11.5 Annexin V stainings, E11.5 Fluidigm sorting
CD45 PE-CF594	30-F11	BD	E10.5 FL analysis
CD45.1 APC	A20	BD	Repopulation analysis, BM LSK reconstitution analysis
CD45.2 FITC	104	eBioscience	Repopulation analysis, BM LSK reconstitution analysis
CD48 APC	HM48-1	BioLegend	E12.5-E14.5 FL LSK staining
CD48 PE	HM48-1	eBioscience	BM LSK reconstitution analysis
CD71 PE	RI7217	BioLegend	E12.5-E14.5 FL Erythroid staining, E11.5 FL analysis, E10-E11.5 EMP Blood analysis, E12.5 FL imaging flow cytometry
CD8a PE-Cy7	53-6.7	eBioscience	Repopulation analysis
CD8a PE-Cy5	53-6.7	BD	Lineage cocktail for BM LSK reconstitution analysis, Lineage cocktail for E12.5 FL analysis
Dlk1 FITC	24-11	MBL	E10.5-E12.5 FL analysis
DRAQ5		eBioscience	DNA stain for imaging flow cytometry
F4/80 AlexaFluor488	BM8	BioLegend	E11.5 and E14.5 macrophage staining
F4/80 APC	BM8	BioLegend	E11.5 LMPP staining
F4/80 PE-Cy5	BM8	BioLegend	Lineage cocktail for BM LSK reconstitution analysis, Lineage cocktail for E12.5-E14.5 FL LSK staining, Lineage cocktail for E12.5 FL analysis, Lineage cocktail for Fluidigm sorting (LMPP staining)
Flt3 Biotin	A2F10	BioLegend	E11.5 LMPP staining
Flt3 BV421	A2F10	BD	E11.5 Fluidigm sorting
Gr1 (Ly6G) APC	RB6-8C5	eBioscience	E11.5 LMPP staining
Gr1 (Ly6G) APC-Cy7	RB6-8C5	BD	Repopulation analysis
Gr1 (Ly6G) Biotin	RB6-8C5	BD	E12.5-E14.5 FL Erythroid staining lineage cocktail
Gr1(Ly6G) PE-Cy5	RB6-8C5	BioLegend	Lineage cocktail for BM LSK reconstitution analysis, Lineage cocktail for E12.5-E14.5 FL LSK staining, Lineage cocktail for E12.5 FL analysis, Lineage cocktail for Fluidigm sorting (LMPP staining)
Hoechst 33258	-	Sigma	Viability dye
IL7R PE	A7R34	Thermo Fisher	E11.5 LMPP staining, E11.5 Fluidigm sorting
c-Kit AF700	2B8	BD	BM LSK reconstitution analysis
c-Kit APC-ef780	2B8	eBioscience	E11.5 LMPP staining, E12.5-E14.5 FL LSK staining
c-Kit FITC	2B8	eBioscience	E10.5-11.5 AGM Pre-HSC staining, E11.5 RNA-Seq and Fluidigm sorting, E9.5 EMP analysis, E11.5 YS-FL EMP analysis, E11.5 FL analysis, E10.5 AGM, YS analysis, E10-E11.5 EMP Blood analysis

c-Kit PE-Cy7	2B8	BD	E11.5 BrdU, E11.5 Annexin V stainings
c-Kit PE-CF594	2B8	BD	E12.5 FL imaging flow cytometry
NK1.1 APC	PK136	eBioscience	E11.5 LMPP staining
NK1.1 PE-Cy5	PK136	BioLegend	Lineage cocktail for BM LSK reconstitution analysis, Lineage cocktail for E12.5-E14.5 FL LSK staining, Lineage cocktail for E12.5 FL analysis, Lineage cocktail for E11.5 LMPP staining and Fluidigm sorting
Sca1 FITC	E13-161.7	BioLegend	E11.5 LMPP staining
Sca1 Pacific Blue	E13-161.7	BioLegend	E12.5-E14.5 FL LSK staining
Sca1 PECy7	D7	eBioscience	E11.5 Fluidigm sorting
Sca1 V450	D7	BD	BM LSK reconstitution analysis
Streptavidin QDot 655	-	Thermo Fisher	E11.5 LMPP staining
Streptavidin PE-Cy7	-	Thermo Fisher	E12.5-E14.5 FL Erythroid staining lineage cocktail
Ter119 AF488	TER-119	BioLegend	E12.5 FL imaging flow cytometry
Ter119 APC	TER-119	BioLegend	E11.5 LMPP staining
Ter119 APC-ef780	TER-119	eBioscience	E11.5 BrdU, E11.5 Annexin V stainings, E14.5 FL Erythroid staining, E9.5 EMP analysis, E10.5 AGM, YS analysis, E10.5 FL analysis, E11.5 macrophage staining, E11.5 Fluidigm sorting
Ter119 PE-Cy5	TER-119	BioLegend	Lineage cocktail for BM LSK reconstitution analysis, Lineage cocktail for E12.5-E14.5 FL LSK staining, Lineage cocktail for E12.5 FL analysis, Lineage cocktail for Fluidigm sorting (LMPP staining)
Ter119 PE-Cy7	TER-119	BD	E11.5 AGM Pre-HSC staining, RNA-Seq sorting, E11.5 YS-FL EMP analysis, E11.5 FL analysis, E14.5 macrophage staining, E10-E11.5 EMP Blood analysis
VE-Cadherin ef660	eBioBV13	eBioscience	E11.5 BrdU, E11.5 Annexin V stainings, E10.5-E11.5 AGM Pre-HSC staining, E11.5 RNA-Seq and Fluidigm sorting, E10.5 AGM, YS analysis, E10.5-E12.5 FL analysis

Appendix Table S4. Taqman probes used for classic and multiplex qRT-PCR analysis.

Assay name	Full gene name	Assay ID	Application(s)
Atp5a1	ATP synthase, H+ transporting, mitochondrial F1 complex, alpha subunit 1	Mm00431960_m1	Single gene and multiplex qRT-PCR
Bax	BCL2-associated X protein	Mm00432051_m1	Multiplex qRT-PCR
Bcl2	B cell leukemia/ lymphoma 2	Mm00477631_m1	Multiplex qRT-PCR
Bcl2l1	BCL2-like 1	Mm00437783_m1	Multiplex qRT-PCR
Bmi1	Bmi1 polycomb ring finger oncogene	Mm03053308_g1	Multiplex qRT-PCR
BTG1	B cell translocation gene 1, anti-proliferative	Mm02391761_m1	Multiplex qRT-PCR
Fos	FBJ osteosarcoma oncogene	Mm00487425_m1	Multiplex qRT-PCR
ccnd1	Cyclin D1	Mm00432359_m1	Multiplex qRT-PCR
ccnd2	Cyclin D2	Mm00438070_m1	Multiplex qRT-PCR
Cdkn1a	cyclin-dependent kinase inhibitor 1A (p21)	Mm04205640_g1	Multiplex qRT-PCR
Cdkn1b	cyclin-dependent kinase inhibitor 1B (p27)	Mm00438168_m1	Multiplex qRT-PCR
Ezh2	enhancer of zeste homolog 2	Mm00468464_m1	Multiplex qRT-PCR
Hes1	hairy and enhancer of split 1	Mm01342805_m1	Multiplex qRT-PCR
HIF-1a	hypoxia inducible factor 1, alpha subunit	Mm00468869_m1	Multiplex qRT-PCR
Hprt1	hypoxanthine guanine phosphoribosyl transferase	Mm01545399_m1	Multiplex qRT-PCR
Kit	Kit oncogene	Mm00445212_m1	Multiplex qRT-PCR
Kitl	Kit ligand	Mm00442972_m1	Single gene qRT-PCR
Lnk	SH2B adaptor protein 3; alias Lnk	Mm00493162_g1	Multiplex qRT-PCR
Myc	myelocytomatosis oncogene	Mm00487804_m1	Multiplex qRT-PCR
Notch2	Notch2	Mm00803077_m1	Multiplex qRT-PCR
Rcan1	regulator of calcineurin 1	Mm01213406_m1	Multiplex qRT-PCR
Stat5a	signal transducer and activator of transcription 5A	Mm03053818_s1	Multiplex qRT-PCR
Ubc	ubiquitin C	Mm01201237_m1	Multiplex qRT-PCR

Appendix Table S5. List of genes used in multiplex qRT-PCR analysis and references.

Gene name	Function	Known cell type(s)	Reference(s)	Previously observed effect(s) and additional information
Atp5a1	Housekeeper	-	-	-
Bax	Apoptosis (pro-apoptotic)	Primordial germ cells (PGCs)	[2]	Upregulation in absence of Kitl
Bcl2	Apoptosis (anti-apoptotic)	NK cells, erythroid cells, HSCs, mast cells	[6-9]	Downregulation in absence of Kitl; upregulation in response to Kitl
Bcl2l1	Apoptosis (anti-apoptotic)	Erythroid cell lines, mast cells	[7]	Downregulation in absence of Kitl
Bmi1	Epigenetic	HSCs	[10]	Downregulation in absence of Kitl
BTG1	Proliferation	Erythroid cells	[11,12]	Repressed by Kitl. PI3k/Akt/Foxo3a target; negative regulator of cell proliferation
Fos	Proliferation, differentiation and survival	Porcine aortic endothelial cells	[13]	Upregulation in response to Kitl Downstream of PI3k/Akt
Myc	Proliferation, differentiation and survival	Erythroblasts, BM HSCs	[14-16]	Upregulation in response to Kitl. Downstream target of Kitl/Kit via MEK/ERK and PI3K/Akt
Cdkn1a (p21)	Cell cycle (Cyclin dependent kinase inhibitor)	Myeloid cell lines, erythroblasts, hematopoietic cells in vivo	[17-19]	Upregulation/downregulation in response to Kitl (different reports)
Cdkn1b (p27)	Cell cycle inhibitor	Myeloid cell lines, hematopoietic cells in vivo	[17,18]	Downregulation in response to Kitl.
Cyclin D1	Cell cycle	Multiple cell types	[17,20]	Potential upregulation in response to Kitl.
Cyclin D2	Cell cycle	Multiple cell types	[17,20]	Potential upregulation in response to Kitl.
Ezh2	Epigenetic	HSCs	[10]	Downregulation in absence of Kitl. Involved in HSC function and erythropoiesis
Hes1	Notch Signaling	Primary erythroblasts	[9]	Upregulation in response to Kitl.
HIF-1a	Hematopoietic function	Hematopoietic cell lines (M-07e, Kasumi1, Ba/F3)	[21]	Upregulation in response to Kitl. Downstream target (Akt/mTOR), also involved in hematopoiesis and erythropoiesis in the embryo
Hprt	Housekeeper	-	-	-
Lnk	Adaptor protein; hematopoietic function	AGM hematopoietic cells, HSCs, osteoblasts	[10,22,23]	Downregulation in absence of Kitl. Broad inhibitor of growth factor and cytokine signaling; negative regulator of hematopoiesis in the AGM.
Notch2	Notch Signaling	Primary erythroblasts	[9]	Upregulation in response to Kitl
Rcan1	Calcineurin/ NF-kB inhibitor	Bone marrow derived mast cells	[24]	Upregulation in response to Kitl.
Stat5a	Hematopoietic function, signal transduction	Erythroid cell lines	[7,25]	Upregulation in response to Kitl. Dobule Stat5a and 5b knockout results in severe anemia
Ubc	Housekeeper	-	-	-

Supplemental references

1. Buono M, Facchini R, Matsuoka S, Thongjuea S, Waithe D, Luis TC, Giustacchini A, Besmer P, Mead AJ, Jacobsen SE, *et al.* (2016) A dynamic niche provides Kit ligand in a stage-specific manner to the earliest thymocyte progenitors. *Nature cell biology* **18:** 157-167
2. Runyan C, Schaible K, Molyneaux K, Wang Z, Levin L, Wylie C (2006) Steel factor controls midline cell death of primordial germ cells and is essential for their normal proliferation and migration. *Development* **133:** 4861-4869
3. Sato T, Yokonishi T, Komeya M, Katagiri K, Kubota Y, Matoba S, Ogonuki N, Ogura A, Yoshida S, Ogawa T (2012) Testis tissue explantation cures spermatogenic failure in c-Kit ligand mutant mice. *Proceedings of the National Academy of Sciences of the United States of America* **109:** 16934-16938
4. Kisanuki YY, Hammer RE, Miyazaki J, Williams SC, Richardson JA, Yanagisawa M (2001) Tie2-Cre transgenic mice: a new model for endothelial cell-lineage analysis in vivo. *Developmental biology* **230:** 230-242
5. Ding L, Saunders TL, Enikolopov G, Morrison SJ (2012) Endothelial and perivascular cells maintain haematopoietic stem cells. *Nature* **481:** 457-462
6. Carson WE, Haldar S, Baiocchi RA, Croce CM, Caligiuri MA (1994) The c-kit ligand suppresses apoptosis of human natural killer cells through the upregulation of bcl-2. *Proceedings of the National Academy of Sciences of the United States of America* **91:** 7553-7557
7. Kapur R, Zhang L (2001) A novel mechanism of cooperation between c-Kit and erythropoietin receptor. Stem cell factor induces the expression of Stat5 and erythropoietin receptor, resulting in efficient proliferation and survival by erythropoietin. *The Journal of biological chemistry* **276:** 1099-1106
8. Thoren LA, Liuba K, Bryder D, Nygren JM, Jensen CT, Qian H, Antonchuk J, Jacobsen SE (2008) Kit regulates maintenance of quiescent hematopoietic stem cells. *Journal of immunology* **180:** 2045-2053
9. Zeuner A, Francescangeli F, Signore M, Venneri MA, Pedini F, Felli N, Pagliuca A, Conticello C, De Maria R (2011) The Notch2-Jagged1 interaction mediates stem cell factor signaling in erythropoiesis. *Cell death and differentiation* **18:** 371-380
10. Kent DG, Dykstra BJ, Cheyne J, Ma E, Eaves CJ (2008) Steel factor coordinately regulates the molecular signature and biologic function of hematopoietic stem cells. *Blood* **112:** 560-567
11. Bakker WJ, Blazquez-Domingo M, Kolbus A, Besooyen J, Steinlein P, Beug H, Coffer PJ, Lowenberg B, von Lindern M, van Dijk TB (2004) FoxO3a regulates erythroid differentiation and induces BTG1, an activator of protein arginine methyl transferase 1. *The Journal of cell biology* **164:** 175-184
12. Bakker WJ, van Dijk TB, Parren-van Amelsvoort M, Kolbus A, Yamamoto K, Steinlein P, Verhaak RG, Mak TW, Beug H, Lowenberg B, *et al.* (2007) Differential regulation of Foxo3a target genes in erythropoiesis. *Molecular and cellular biology* **27:** 3839-3854
13. Lennartsson J, Blume-Jensen P, Hermanson M, Ponten E, Carlberg M, Ronnstrand L (1999) Phosphorylation of Shc by Src family kinases is necessary for stem cell factor receptor/c-kit mediated activation of the Ras/MAP kinase pathway and c-fos induction. *Oncogene* **18:** 5546-5553
14. Munugalavadla V, Dore LC, Tan BL, Hong L, Vishnu M, Weiss MJ, Kapur R (2005) Repression of c-kit and its downstream substrates by GATA-1 inhibits cell proliferation during erythroid maturation. *Molecular and cellular biology* **25:** 6747-6759
15. Munugalavadla V, Kapur R (2005) Role of c-Kit and erythropoietin receptor in erythropoiesis. *Critical reviews in oncology/hematology* **54:** 63-75

16. Zhang Z, Zhu P, Zhou Y, Sheng Y, Hong Y, Xiang D, Qian Z, Mosenson J, Wu WS (2017) A novel slug-containing negative-feedback loop regulates SCF/c-Kit-mediated hematopoietic stem cell self-renewal. *Leukemia* **31:** 403-413
17. Kent D, Copley M, Benz C, Dykstra B, Bowie M, Eaves C (2008) Regulation of hematopoietic stem cells by the steel factor/KIT signaling pathway. *Clinical cancer research : an official journal of the American Association for Cancer Research* **14:** 1926-1930
18. Mantel C, Luo Z, Canfield J, Braun S, Deng C, Broxmeyer HE (1996) Involvement of p21cip-1 and p27kip-1 in the molecular mechanisms of steel factor-induced proliferative synergy in vitro and of p21cip-1 in the maintenance of stem/progenitor cells in vivo. *Blood* **88:** 3710-3719
19. Haas N, Riedt T, Labbaf Z, Bassler K, Gergis D, Frohlich H, Gutgemann I, Janzen V, Schorle H (2015) Kit transduced signals counteract erythroid maturation by MAPK-dependent modulation of erythropoietin signaling and apoptosis induction in mouse fetal liver. *Cell death and differentiation* **22:** 790-800
20. Sherr CJ, Roberts JM (2004) Living with or without cyclins and cyclin-dependent kinases. *Genes & development* **18:** 2699-2711
21. Pedersen M, Lofstedt T, Sun J, Holmquist-Mengelbier L, Pahlman S, Ronnstrand L (2008) Stem cell factor induces HIF-1alpha at normoxia in hematopoietic cells. *Biochemical and biophysical research communications* **377:** 98-103
22. Matsumoto T, Ii M, Nishimura H, Shoji T, Mifune Y, Kawamoto A, Kuroda R, Fukui T, Kawakami Y, Kuroda T*, et al.* (2010) Lnk-dependent axis of SCF-cKit signal for osteogenesis in bone fracture healing. *The Journal of experimental medicine* **207:** 2207-2223
23. Nobuhisa I, Takizawa M, Takaki S, Inoue H, Okita K, Ueno M, Takatsu K, Taga T (2003) Regulation of hematopoietic development in the aorta-gonad-mesonephros region mediated by Lnk adaptor protein. *Molecular and cellular biology* **23:** 8486-8494
24. Wu Z, Li Y, MacNeil AJ, Junkins RD, Berman JN, Lin TJ (2013) Calcineurin-Rcan1 interaction contributes to stem cell factor-mediated mast cell activation. *Journal of immunology* **191:** 5885-5894
25. Socolovsky M, Fallon AE, Wang S, Brugnara C, Lodish HF (1999) Fetal anemia and apoptosis of red cell progenitors in Stat5a-/-5b-/- mice: a direct role for Stat5 in Bcl-X(L) induction. *Cell* **98:** 181-191
