# Supplementary material for: Kit ligand has a critical role in mouse yolk sac and aorta–gonad–mesonephros hematopoiesis
Source: EMBO Rep. 2018 Aug 30;19(10):e45477. doi: 10.15252/embr.201745477 (PMC6172468; doi:10.15252/embr.201745477)
Supplement: Supplementary file 2 — Expanded View Figures PDF [file EMBR-19-e45477-s002.pdf]

Expanded View Figures

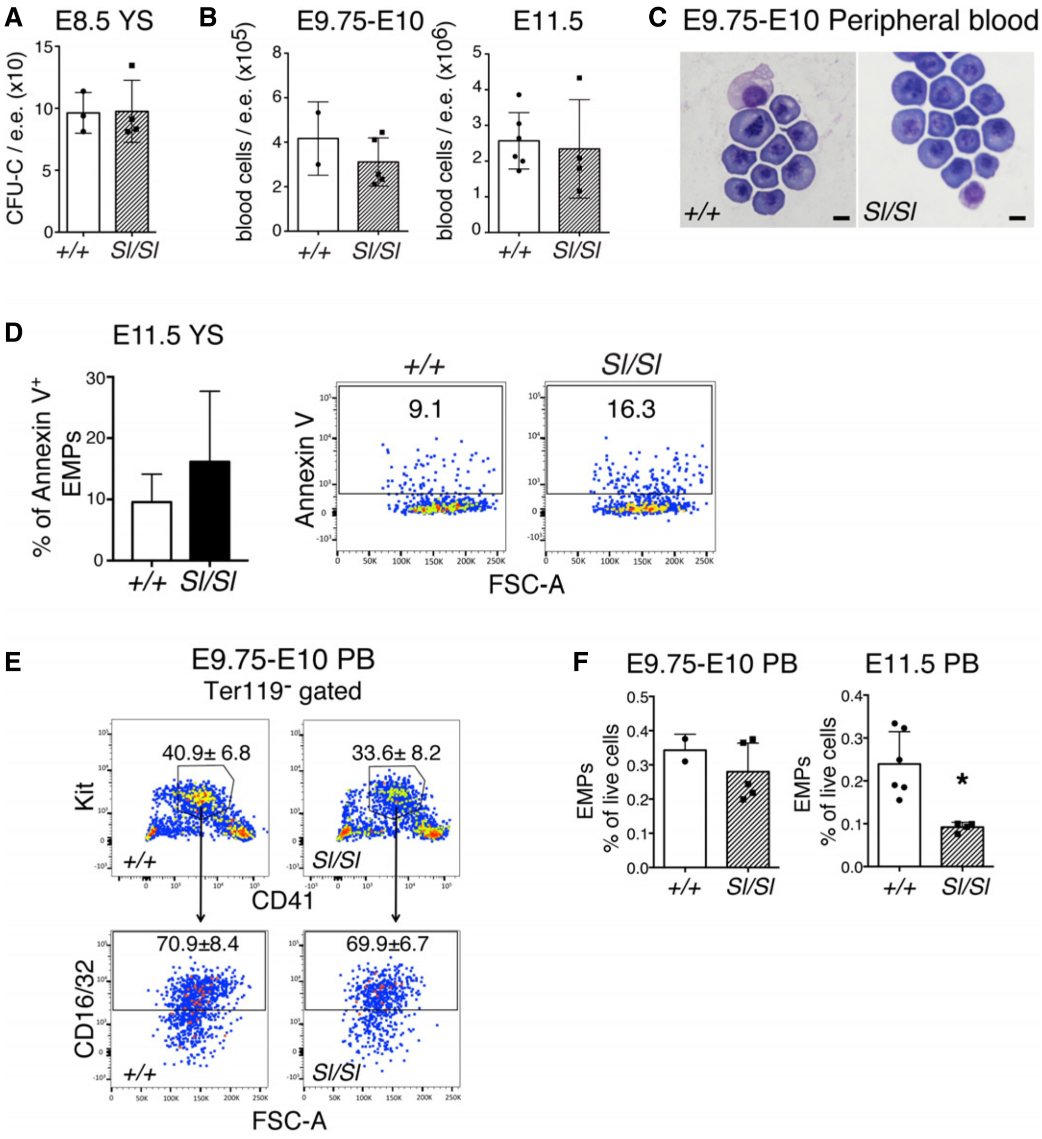

Figure EV1. Kitl is not required for primitive erythropoiesis or YS EMP survival.

A Number of CFU-C in wild type and *Sl/Sl* E8.5 concepti (the head was removed for genotyping). Data (mean ± SD) are from three wild type and four *Sl/Sl* biological replicates (pools of embryos of the same genotype) plated in duplicate. Total number of analyzed embryos: 9 +/+ (3–7 sp), 9 *Sl/Sl* (2–8 sp) over four independent experiments.

B Left: total number of peripheral blood (PB) cells of wild type and *Sl/Sl* E9.75-E10 embryos (28–30 sp, +/+; 27–31, *Sl/Sl*) embryos. *N* = 2 (wild type), *N* = 5 (*Sl/Sl*). Right: total number of PB cells of E11.5 (tail somite range 10–16 (+/+); 12–15 (*Sl/Sl*)) embryos. *N* = 6 (wild type), *N* = 4 (*Sl/Sl*). Error bars represent SD.

C Representative images of Wright-Giemsa stained cytospins from E9.75-E10 (28–30 sp, +/+; 27–31, *Sl/Sl*) wild type and *Sl/Sl* PB. Scale bars: 10 μm. *N* = 2 (wild type), *N* = 5 (*Sl/Sl*).

D Flow cytometry analysis showing the percentage of early apoptotic Annexin-V$^+$ EMPs in wild type and *Sl/Sl* E11.5 YS. EMPs were gated as 7-AAD$^-$ Ter119$^-$ CD41$^+$ Kit$^+$ CD16/32$^+$. Embryos were analyzed in pools of 1–3. *N* = 9 (+/+), *N* = 7 (*Sl/Sl*) biological replicates over three independent experiments. Bar diagrams show mean ± SD values, and FACS plots representative results. Total number of analyzed embryos: 13 (+/+), 9 (*Sl/Sl*). Tail somite range: 11–17 (+/+); 11–17 (*Sl/Sl*).

E Representative dot plots showing the flow cytometric identification of EMPs (Ter119$^-$ Kit$^+$ CD41$^+$ CD16/32$^+$) in peripheral blood (PB) of wild type and *Sl/Sl* midgestation embryos.

F Percentages (mean ± SD) of EMPs in the PB of wild type and Sl/Sl embryos as identified in (E). For E9.75-E10, PB from two wild type (28–30 sp) and five *Sl/Sl* (27–31) individual embryos was analyzed. For E11.5, PB from 1 to 3 embryos was pooled and six wild type and four *Sl/Sl* biological replicates were analyzed, with 10 wild type and 6 *Sl/Sl* embryos analyzed in total. *$P < 0.05$ (unpaired two-tailed Student's *t*-test).

© 2018 The Authors

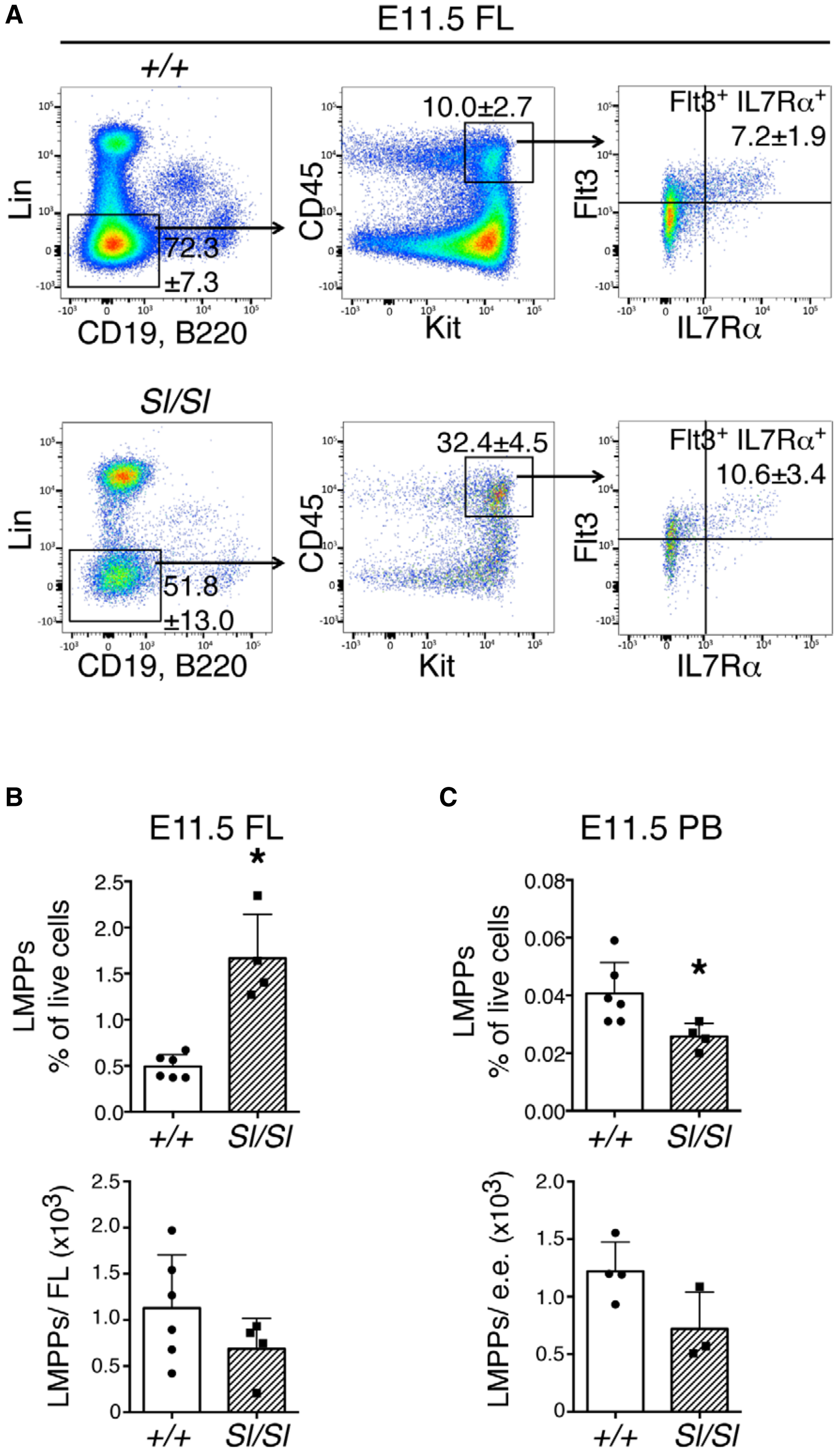

Figure EV2. Lymphomyeloid immune-restricted progenitor (LMPP) numbers are reduced in *Sl/Sl* FL and PB.

A Flow cytometry analysis of Lin^{-} CD19^{-} B220^{-} CD45^{+} Kit^{+} Flt3^{+} IL7Rα^{+} LMPPs in wild type and *Sl/Sl* E11.5 FL. Lineage cocktail (Lin): Ter119, CD3e, F4/80, Nk1.1, Gr1. Gates and percentages within gate are indicated in representative dot plots. Percentages are the mean (± SD) of six wild type and four *Sl/Sl* biological replicates, with each replicate consisting of FL cells from individual or pools of up to three embryos. A total of 10 wild type (10–16 tail sp) and 6 *Sl/Sl* (12–15 tail sp) embryos were analyzed.

B Percentage and number of LMPP per E11.5 FL, as identified by flow cytometry and total FL cell counts. Data are the mean (± SD); samples as in (A).

C Percentage and number of LMPP in PB, as identified by flow cytometry and PB cell counts per embryo. Data are the mean (± SD); samples as in (A). e.e.: embryo equivalent.

Data information: $^{*}P < 0.05$ (unpaired two-tailed Student's *t*-test).

© 2018 The Authors

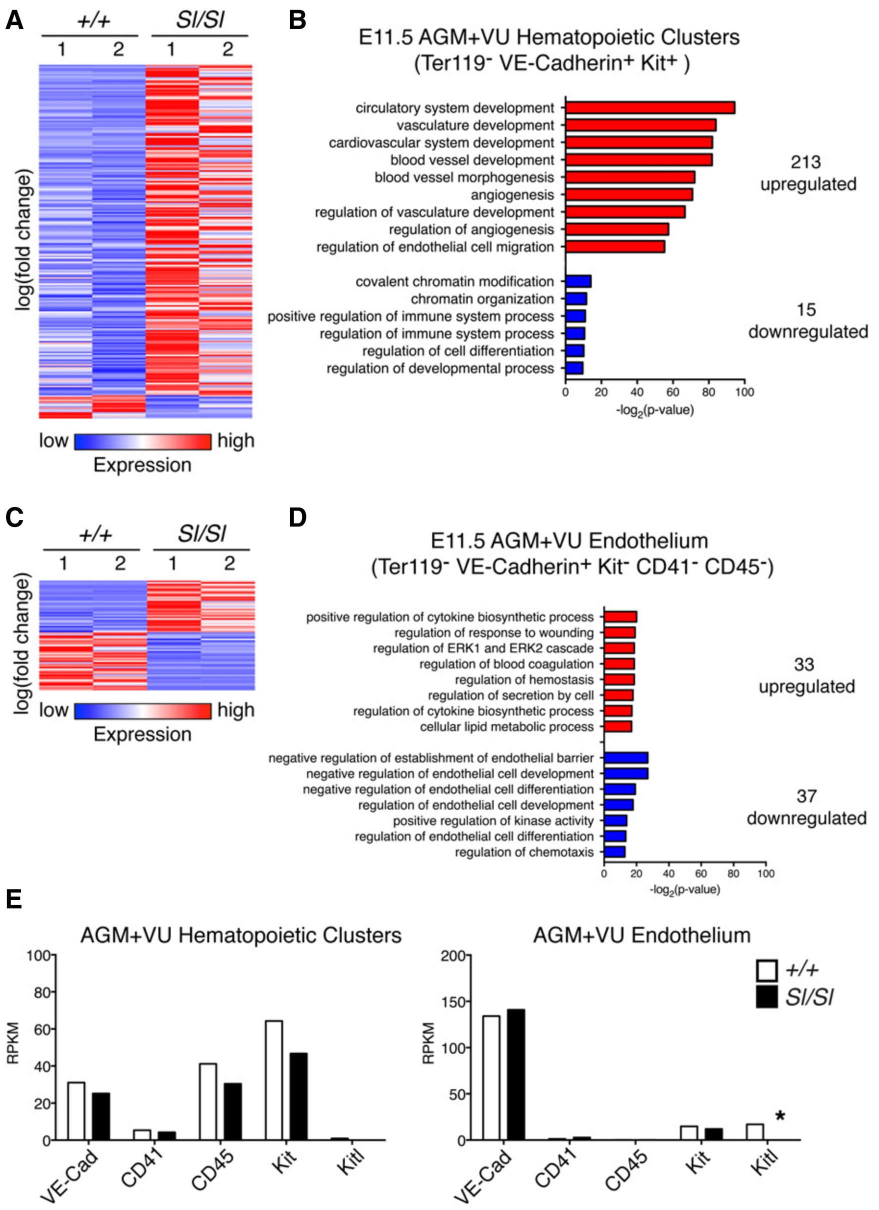

Figure EV3. Transcriptional analysis of wild type and *Sl/Sl* AGM hematopoietic clusters and endothelium.

A E11.5 AGM+VU Ter119$^-$ VE-Cadherin$^+$ Kit$^+$ cells (hematopoietic clusters) were sorted from wild type and *Sl/Sl* embryos (pools of 1–4 embryos of the same genotype; 2 sorting experiments). Duplicate samples of 100 cells each were processed using SMARTSEQ2 [80]. The un-clustered heat map shows 228 genes differentially expressed ($P < 0.05$, FDR < 0.1) between wild type and *Sl/Sl* hematopoietic clusters as detected by RNA-Seq and is ordered by log(fold change) with high positive values on top and negative values on bottom. Each column shows a biological replicate.

B Gene ontology (GO) analysis performed with the MetaCore suite (Thomson Reuters) of the 213 upregulated and 15 downregulated genes identified by RNA-Seq, showing selected categories within the top over-represented GO processes.

C E11.5 AGM+VU Ter119$^-$ VE-Cadherin$^+$ Kit$^-$ CD41$^-$ CD45$^-$ (endothelial cells) were sorted from wild type and *Sl/Sl* embryos (pools of 1–4 embryos of the same genotype; 2 sorting experiments). Duplicate samples of 100 cells each were processed using SMARTSEQ2. The un-clustered heat map shows 70 genes differentially expressed ($P < 0.05$, FDR < 0.1) between wild type and *Sl/Sl* endothelium as detected by RNA-Seq and is ordered by log(fold change) with high positive values on top and negative values on bottom. Each column shows a biological replicate.

D Gene ontology (GO) analysis performed with the MetaCore suite (Thomson Reuters) of the 33 upregulated and 37 downregulated genes identified by RNA-Seq, showing selected categories within the top over-represented GO processes.

E RPKM expression of genes encoding for phenotypic markers used to identify and sort cell populations, in wild type and *Sl/Sl* E11.5 AGM+VU hematopoietic clusters (Ter119$^-$ VE-Cadherin$^+$ Kit$^+$) and endothelium (Ter119$^-$ VE-Cadherin$^+$ Kit$^-$ CD41$^-$ CD45$^-$). Note low expression of Kit in the endothelium, and absence of Kitl in *Sl/Sl* (as expected). *$P < 0.05$ (unpaired two-tailed Student's *t*-test).

 © 2018 The Authors

Figure EV4. Validation and quantification of Kitl-tdTomato reporter expression.

A Kitl-tdTomato⁺ and Kitl-tdTomato⁻ subsets were isolated from pooled E8.5 (3–8 sp) Kitl-tdTomato transgenic concepti and E10.5 (31–36 sp) AGM regions (including vitelline and umbilical arteries) (AGM+VU), YS, and FL. PAS: para-aortic splanchnopleura. Sort gates as indicated. Total number of Kitl-tdTomato transgenic embryos analyzed: 14 (E8.5), 21 (E10.5). *N* = 2 independent experiments for E8.5; *N* = 2 independent experiments for E10.5.

B qRT–PCR analysis of endogenous *Kitl* in the Kitl-tdTomato$^+$ and Kitl-tdTomato$^-$ subsets shown in (A). Endogenous *Kitl* expression (relative to *Atp5a1*) is virtually restricted to Kitl-tdTomato$^+$ cells.

C Control sample for confocal whole-mount immunofluorescence analysis of *Kitl*-tdTomato expression in the E8.5 (6–7 sp) YS BI. The full staining (using CD31, GFP and anti-RFP/tdTomato antibodies) has been performed on a 23GFP embryo. No significant background is observed. A single 2.5-μm-thick *Z*-slice is shown. Scale bar: 50 μm.

D Flow cytometry analysis of E10.5 (32–37 sp) Kitl-tdTomato transgenic YS. Live cells were gated as total Ter119$^-$, or as Ter119$^-$ endothelial cells (EC: VE-Cadherin$^+$ Kit$^-$ CD41$^-$ CD45$^-$), hematopoietic clusters (HC: VE-Cadherin$^+$ Kit$^+$), hematopoietic progenitors (HP: VE-Cadherin$^-$ Kit$^+$), and mesenchymal cells (MC: VE-Cadherin$^-$ Kit$^-$ CD41$^-$ CD45$^-$). Representative dot plots are shown. Nineteen Kitl-tdTomato transgenic embryos were analyzed (pooled tissues) in a total of three independent experiments. Data in the graph are mean (± SD).

E Confocal whole-mount immunofluorescence analysis of a E9.5 (25–26 sp) Kitl-tdTomato embryo. Left image shows a maximum intensity 3D projection from a 500-μm-thick *Z*-stack. The region indicated in the boxed inset is magnified in the middle and right panels, which show a single 2.5-μm-thick longitudinal slice. Arrowheads indicate Kitl-tdTomato$^+$ CD31$^+$ endothelial cells in the ventral wall of the dorsal aorta. Scale bars: 150 μm (3D projection); 50μm (slice inset). va: vitelline artery; da: dorsal aorta. *N* = 3 embryos analyzed.

F Flow cytometry analysis of E10.5 Kitl-tdTomato transgenic AGM+VU from the same embryos as in (D). Representative dot plots are shown. Live cells were gated as Ter119$^-$ or as Ter119$^-$ EC, HC, MC, as shown. Data in the graph are mean (± SD).

G Flow cytometry analysis of Kitl-tdTomato expression in the non-hematopoietic (Ter119$^-$ CD41$^-$ CD45$^-$) components of the E10.5 (32–37 sp) FL. Endothelial (EC: VE-Cadherin$^+$ Dlk1$^-$), mesenchymal (MC: VE-Cadherin$^-$ Dlk1$^-$) cells, and hepatoblasts (HB: VE-Cadherin$^-$ Dlk1$^+$) were gated as indicated. Representative dot plots with percentage of Kitl-TdTomato$^+$ cells are shown. HB make up the majority of the non-hematopoietic FL cells and, in line with previous reports [65,66], show extensive Kitl expression. A total of 19 Kitl-tdTomato embryos, pooled according to genotype, were analyzed over three independent experiments (*N* = 2 experiments for MC and HB. Kitl-tdTomato$^+$ range: MC 23.1–40.4%; HB 84.1–86.9%). Data in the graph are mean (± SD).

© 2018 The Authors

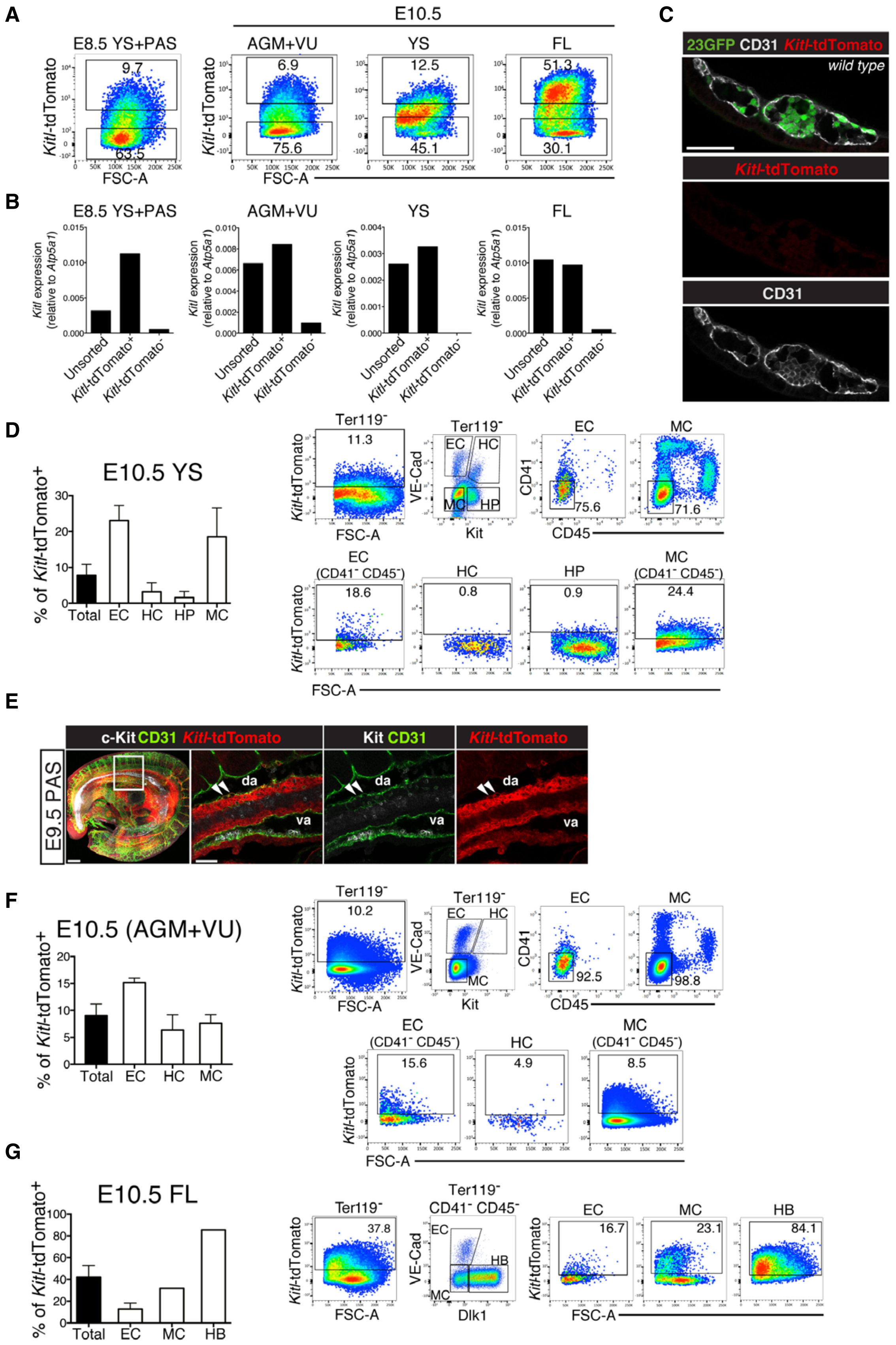

Figure EV4.

© 2018 The Authors

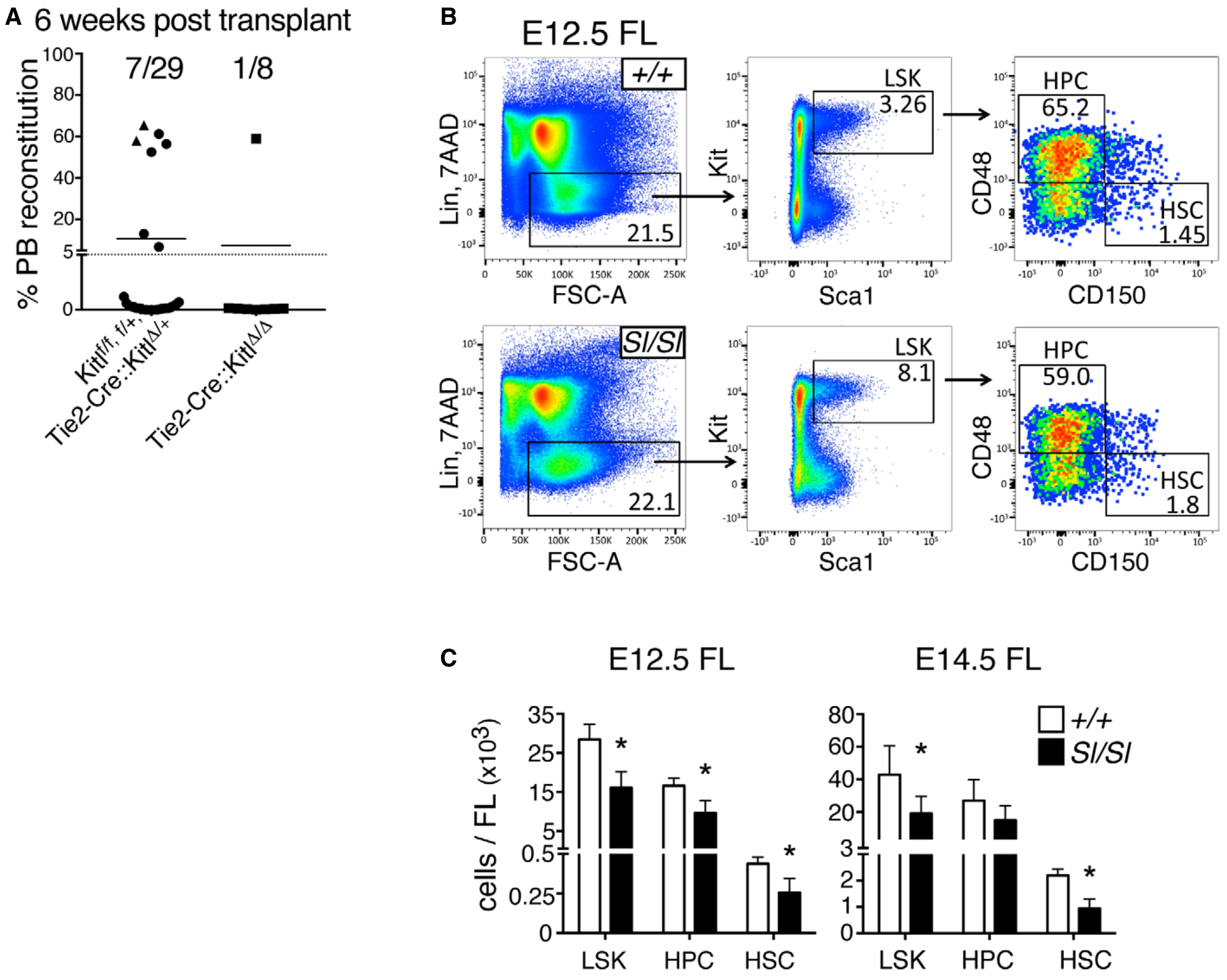

Figure EV5. Effects of Kitl loss on AGM and FL HSCs.

A Analysis of long-term multi-lineage HSC potential in E11.5 Tie2Cre::*Kitl*$^{\Delta/\Delta}$ and control Tie2Cre::*Kitl*$^{\Delta/+}$ or *Kitl*$^{f/f \text{ or } f/+}$AGM+VU. Irradiated CD45.1 syngeneic mice were transplanted with 1 e.e. of AGM+VU cells. PB chimerism is represented as the percentage of donor CD45.2^{+} cells among total CD45^{+} cells, 6 weeks after transplant. A total of 29 recipients were transplanted with Tie2Cre::*Kitl*$^{\Delta/+}$ or *Kitl*$^{f/f \text{ or } f/+}$ cells and 8 with Tie2Cre::*Kitl*$^{\Delta/\Delta}$ cells, over seven independent experiments. *Kitl*$^{f/f \text{ or } f/+}$ is represented with a circle, Tie2Cre::*Kitl*$^{\Delta/+}$ a triangle and Tie2Cre::*Kitl*$^{\Delta/\Delta}$ with a square. Tail somite range: 12–17 (control); 12–17 (Tie2Cre::*Kitl*$^{\Delta/\Delta}$).

B Analysis of phenotypic-defined HSPCs populations in wild type and *Sl/Sl* E12.5 FL. Representative dot plots with gates and percentages of 7-AAD^{-} Lin^{-} (F4/80^{-} CD3e^{-} Nk1.1^{-} Ter119^{-} Gr-1^{-} B220^{-} CD19^{-}) LSK, HPC, and HSC are shown. Fetal livers were analyzed individually. *N* = 4 (wild type); *N* = 5 (Sl/Sl).

C Number of LSK, HPC, and HSC per FL, determined by flow cytometry in (E) and total FL cell counts. Number of FL analyzed at E12.5: as in (D). E14.5 data are the mean (± SD) of five biological replicates for either genotype. Replicates consisted of cells from 1 to 3 FLs, with a total of 10 wild type and 5 Sl/Sl FLs analyzed. $^{*}P < 0.05$ (unpaired two-tailed Student's *t*-test).

© 2018 The Authors
